# Comparative analysis of facial aesthetics in AI generated versus conventionally crafted digital smile designs—a cross-sectional study

**DOI:** 10.1038/s41405-025-00367-z

**Published:** 2025-09-15

**Authors:** Kriti Kaushik, Ann Sales, Shobha J. Rodrigues

**Affiliations:** Department of Prosthodontics, Manipal College of Dental Science Mangalore, Manipal Academy of Higher Education, Manipal, India

**Keywords:** Fixed prosthodontics, Veneers

## Abstract

**Aim:**

This study aimed to evaluate the aesthetic preferences of traditional digital smile designs and artificial intelligence (AI)-generated smile designs among dentists, dental students, and laypersons, addressing gaps in previous research on the clinical acceptability of AI in prosthodontic aesthetics.

**Materials and methods:**

A cross-sectional, questionnaire-based study was conducted via an online survey distributed across India between 2024 and 2025. A total of 320 participants, including dental students, dentists, and nondental professionals, were recruited on the basis of calculated sample size requirements. Smile designs were created for four clinical cases via Exo-CAD software, employing two methods: conventional manual design by prosthodontists and AI-based automated design. The participants evaluated paired smile designs and indicated their aesthetic preferences. Demographic data were also collected. Chi-square (χ²) tests were applied for statistical analysis, with a significance level set at *p* < 0.05.

**Results:**

No significant differences in aesthetic preferences were observed based on sex, age, or occupation. Overall, manually crafted smile designs were consistently preferred across all the participant categories. However, AI-generated smiles for Cases 3 and 4 presented relatively higher acceptance rates (39.4% and 39.7%, respectively) than those for Cases 1 and 2 did. The findings suggest that while AI algorithms can achieve acceptable levels of aesthetic appeal, they still lack the human touch essential for capturing nuanced facial dynamics and emotional context.

**Conclusion:**

Although AI-based smile design systems demonstrate promise in improving workflow efficiency and consistency, they are currently unable to replicate the individualized artistic judgment of experienced clinicians. Manual intervention remains critical for achieving truly personalized and aesthetically harmonious outcomes. Future approaches should consider hybrid models that combine AI automation with clinician-led customization to increase both the efficiency and patient satisfaction of smile aesthetics.

## Introduction

In contemporary dentistry, facial aesthetics are vital because they have a large impact on psychological and functional health. Professional success, social confidence, and self-esteem are associated with having an attractive smile [1]. Smile design is a crucial component of prosthodontics and cosmetic dentistry that requires careful consideration of tooth morphology, gingival aesthetics, facial proportions, and symmetry [2]. The practice of enhancing a person’s smile through different dental processes while utilizing the concepts of harmony, symmetry, and proportion to produce an aesthetically pleasing result is known as “smile design.” This technique is used to address issues such as misalignment, discolouration, and disproportionate tooth size to improve the appearance and functionality of teeth. Wax-ups, cephalometric analysis, and photographic evaluations were the mainstays of traditional smile design techniques in the past. These techniques are subjective and heavily reliant on the clinical skill of the practitioner [3, 4]. Computer-aided design (CAD) and artificial intelligence (AI)-based smile design tools emerged as a result of digital dental advancements, providing automated, accurate, and standardized processes [5, 6]. Among the most recent developments are AI-driven smile design algorithms that create personalized smile designs in real time by analysing occlusion patterns, lip dynamics, and facial landmarks [7]. Although there are still concerns about the aesthetic acceptability of AI-generated smiles in comparison with traditional techniques, these AI-based solutions offer increased efficiency [8]. Critics say that human creative judgment is essential in producing natural, patient-specific smiles despite AI proponents stating that it improves precision and decreases chair-side time [9, 10]. From conventional artistic methods to computer advances that incorporate both two-dimensional (2D) and three-dimensional (3D) simulations, smile design techniques have experienced tremendous evolution. In the past, cephalometric rules, golden proportions, and artistic ideas served as the foundation for manual smile design [11]. The golden ratio was first proposed by Levin et al. [5]. as a benchmark for dental aesthetics [12]. Other studies have concentrated on the importance of symmetry, facial proportions, and occlusal harmony in attaining the best possible aesthetic results [13, 14]. By incorporating 2D and 3D digital simulations into treatment planning, digital smile design (DSD) software transformed prosthodontics in 2008 and enabled dentists to visualize possible outcomes prior to treatment, thereby involving patients in the decision-making process [15]. Although DSD has greatly increased predictability and accuracy, it is still a laborious procedure requiring a high level of technical knowledge [9, 16]. With features including automated picture identification, predictive analytics, and virtual treatment simulations, artificial intelligence (AI) applications in digital dentistry have grown significantly in recent years [17]. Deep learning algorithms trained on large datasets of visually pleasant smiles are used in AI-driven smile design to automatically identify the optimal tooth proportions and facial harmony [18]. AI also significantly reduces manual errors and chair-side time, increasing workflow efficiency and enhancing predictive modelling for treatment results [2, 19]. There are currently a number of AI-based smile design platforms in use, such as Smilecloud, a deep learning-powered aesthetic modelling system; ExoCAD, a CAD-based program that integrates AI for virtual simulations; and DSD Software, which uses facial analysis to automatically generate smile designs [13, 20, 21]. Notwithstanding these developments in technology, nothing is known about the clinical acceptability of AI-generated smile designs. Although AI promotes efficiency and standardization, little is known about whether AI-generated designs meet the arbitrary aesthetic standards of patients and dentists [17]. A critical examination of previous studies on smile aesthetics and digital smile design revealed several methodological shortcomings and biases, such as a small sample size, a subjective aesthetic rating, no longitudinal follow-up, and few direct comparisons. Professional bias is introduced by the fact that many studies ignore the preferences of laypeople and regular dental patients in favour of focusing solely on dentists and specialists [22]. Furthermore, the majority of studies use nonstandardized rating measures to assess aesthetics, which can result in inconsistent findings and a range of results [23]. The absence of longitudinal follow-up is another significant drawback, since few studies have evaluated patient satisfaction with AI-generated designs over lengthy time periods, making it challenging to ascertain their efficacy and long-term acceptability [24]. Furthermore, a comprehensive grasp of how AI-based systems perform in comparison with traditional digital smile design (DSD) methodologies is hindered by the noteworthy lack of large-scale research that directly compares AI-generated and manually made smiles [25]. For example, in an online survey comparing AI-generated and human-designed smiles, Gülsüm Ceylan et al. (2023) reported that while AI-generated smiles were favoured in symmetrical circumstances, gender, education, and experience had no discernible effect on preference [19]. However, because AI lacks human creative intuition and emotional intelligence, which are frequently essential for attaining individualized aesthetic results, Jain et al. [18] reported that patient satisfaction was greater for traditional DSD procedures. Concerns regarding AI’s capacity to evaluate real-world facial dynamics and its possible limitations in producing naturally harmonious results were also raised by Omar et al. [2], who emphasized that aesthetic success depends not only on dental proportions but also on facial integration. To ascertain the actual aesthetic value of AI in smile design, a carefully monitored study contrasting AI-generated and manually created smile designs is needed in light of these methodological flaws and knowledge gaps. The purpose of this study was to assess the subjective preferences of dentists, dentistry students, and the general public by comparing the aesthetic assessments of artificial intelligence-generated and traditionally created digital smile designs. Through the use of a large, diverse sample and the filling of gaps in prior research, this study will offer important insights into the practical application of AI in digital prosthodontics.

## Materials and methods

### Study overview

The purpose of this cross-sectional study was to evaluate the aesthetic appeal of digital smile designs produced traditionally vs those produced via artificial intelligence (AI)-generated techniques. The study assessed aesthetic preferences across various professional and nonprofessional categories via a questionnaire approach. The results of a prior investigation by Gülsüm Ceylan et al. [19]. were used to calculate the necessary sample size. Using the method for two proportions, the required number of participants was determined via proportions of 44.6% vs 55.7%, an alpha error of 5%, and a power of 80%. To attain statistical validity, the study needed an initial sample of 319 participants.

This study was cross-sectional questionnaire-based research conducted through an online survey distributed across India. The study was designed to assess subjective aesthetic preferences for AI-generated and conventionally crafted smile designs. Data collection took place over a three-month period from 2024 to 2025.

### Participants and group allocation

Inclusion criteria—Dentists, dental students (who have entered clinical training), and laypersons (other professionals)

Exclusion criteria—Individuals under the age of 18 years, individuals with intellectual disabilities, and individuals with visual impairments affecting their ability to assess images

Participants were recruited through purposive sampling via academic, dental, and professional networks across India. Three key demographic groups were recruited: dentists, dental students (in clinical training), and laypersons from various professional backgrounds. The inclusion criteria ensured a balanced representation across age and experience levels. All participants were required to have normal or corrected-to-normal vision to evaluate smile aesthetics accurately.

### Smile design process

The study utilized Exo-CAD, a digital smile design program, to create smile designs for comparative analysis. Four clinical cases were carefully selected to represent diverse facial and dental characteristics, with an emphasis on standardization by choosing an ovoid facial form on the basis of the Leon Williams classification. All the clinical cases involved female subjects to maintain standardization in facial form and reduce confounding variables related to sex-based facial aesthetics. Since female facial aesthetic norms are more extensively documented in the literature, they were chosen to ensure comparability with prior research. The selection process was conducted through a group discussion involving experienced cosmetic dentistry professionals to ensure a balanced representation of clinical scenarios. The clinical cases were selected and reviewed by subject experts, defined as prosthodontists with a minimum of 5 years of clinical experience and specialized training in esthetic dentistry and smile design. All the images were captured in full-face view under consistent lighting and background conditions.

**Fig. 1:**
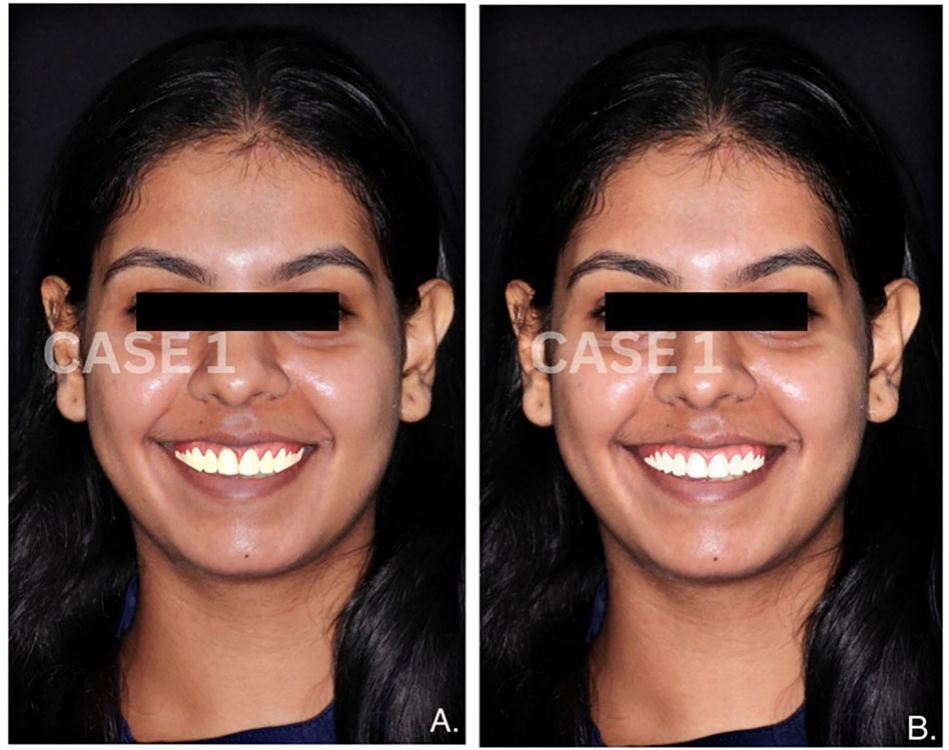
Comparative images of smile designs for Case 1. **A** Smile designed manually by a clinician. **B** Smile generated using AI-based smile design software.

**Fig. 2:**
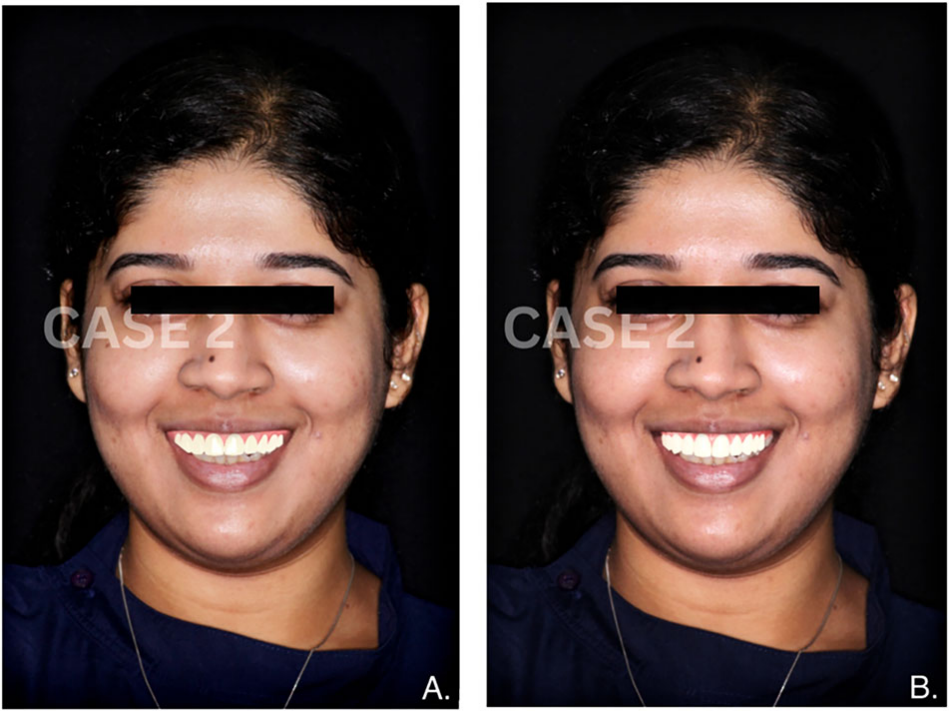
Comparative images of smile designs for Case 2. **A** Smile designed manually by a clinician. **B** Smile generated using AI-based smile design software.

**Fig. 3:**
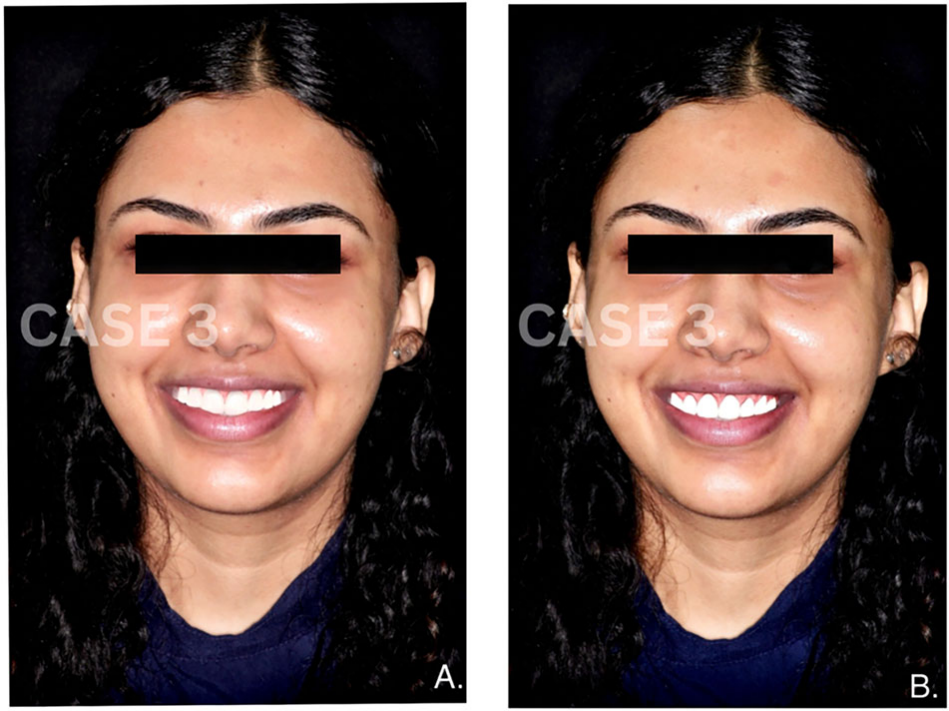
Comparative images of smile designs for Case 3. **A** Smile designed manually by a clinician. **B** Smile generated using AI-based smile design software.

**Fig. 4:**
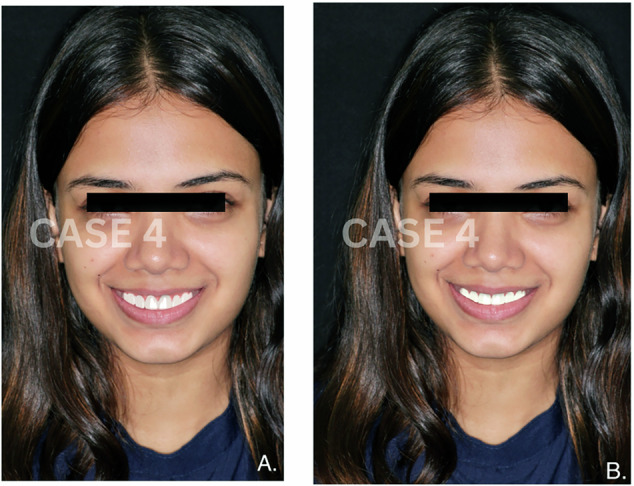
Comparative images of smile designs for Case 4. **A** Smile designed manually by a clinician. **B** Smile generated using AI-based smile design software.

The smile design methodology involves two distinct approaches: conventional digital smile design (DSD) and AI-generated smile design. In the conventional method, a prosthodontist with experience in smile design manually adjusts aesthetic parameters via Exo-CAD software, applying expertise to create an ideal smile. In contrast, the AI-generated smile designs were produced automatically via an AI-based algorithm integrated into the Exo-CAD system. Each of the four cases was designed via both methods, allowing for a direct paired comparison to assess aesthetic preferences. (Figs. 1–4)

A structured online survey was created with Google Forms. The questionnaire was validated by a panel of three senior faculty members from the Department of Prosthodontics who have extensive experience in survey design and smile esthetics. Content validity and clarity were confirmed through iterative feedback and pilot testing. The questionnaire was sent to a wide range of people to obtain participant input. In the first section, participants were asked to provide demographic data, such as age, gender, and occupation; in the second, they were asked about their experience with smile design, including years of experience and familiarity with smile design software; and in the third, they were asked to choose which of the paired images of artificial intelligence (AI)-generated and traditionally crafted smile designs they thought was more aesthetically pleasing. All the answers were anonymized to protect privacy.

The evaluation of aesthetics was based on subjective perception, where participants compared paired smile designs and selected the more aesthetically pleasing option. Although visual attractiveness is subjective, each smile design pair was standardized to present identical facial images, differing only in smile simulation (AI vs manual). The parameters that were implicitly assessed included tooth symmetry, proportion, midline alignment, incisal edge design, gingival display, overall harmony with the facial profile and natural appearance.

The data collected through the online questionnaire were compiled in Microsoft Excel (2023) and imported into SPSS (Version 20) for analysis. Descriptive statistics were used to summarize participant demographics and preferences. The chi-square test (χ²) was used to evaluate associations between categorical variables such as occupation, age, sex, and smile design preference. This nonparametric test was chosen because it is appropriate for evaluating relationships between independent categorical variables without the assumption of a normal distribution. Statistical significance was determined at *p* < 0.05.

### Ethics approval

Ethical considerations were thoroughly addressed before the study was conducted. This study was approved by the Institutional Ethics Committee of the Manipal College of Dental Sciences, Mangalore, a constituent of the Manipal Academy of Higher Education, with approval no. [24151]. The participants provided digital informed consent before taking part in the survey, confirming their voluntary participation. Data confidentiality was strictly maintained, and all images used in the study were anonymized prior to analysis to protect participant privacy. Written informed consent was obtained from all individuals for the publication of their photographs in this manuscript.

## Results

The demographic details and aesthetic preferences of participants are shown in Table 1.

**Table 1:** Demographic details and aesthetic preferences of participants.

		Count	Column N %
Age:	18–25	161	50.3%
	26–35	91	28.4%
	36–45	25	7.8%
	46–55	30	9.4%
	Above 55	13	4.1%
Occupation	Dental Student	98	30.6%
	Dentist	112	35.0%
	Others	110	34.4%
Sex:	Female	192	60.0%
	Male	128	40.0%
Select the smile design you find more aesthetically pleasing in CASE:1	AI preferred	100	31.2%
	Manual Preferred	220	68.8%
Select the smile design you find more aesthetically pleasing in CASE:2	AI Preferred	98	30.6%
	Manual Preferred	222	69.4%
Select the smile design you find more aesthetically pleasing in CASE:3	AI Preferred	126	39.4%
	Manual Preferred	194	60.6%
Select the smile design you find more aesthetically pleasing in CASE:4	AI Preferred	127	39.7%
	Manual Preferred	193	60.3%
% preference to AI	0	80	25.0%
	25	89	27.8%
	50	111	34.7%
	75	20	6.2%
	100	20	6.2%

Table 2 shows the results of the statistical analysis performed via the chi-square test.

**Table 2:** Chi Square Test to compare aesthetic preferences across different professional groups, including dentists, dental students, and laypersons.

	Categories	*N*	Occupation			Chi square	*P* value
			Dental Student (*N* (%))	Dentist (*N* (%))	Others (*N* (%))		
Age:	18–25	161	85 (86.7)	38 (33.9)	38 (34.5)	99.618	**<0.001**
	26–35	91	13 (13.3)	44 (39.3)	34 (30.9)		
	36–45	25	0 (0)	18 (16.1)	7 (6.4)		
	46–55	30	0 (0)	9 (8)	21 (19.1)		
	Above 55	13	0 (0)	3 (2.7)	10 (9.1)		
Sex:	Female	192	55 (56.1)	76 (67.9)	61 (55.5)	4.442	0.109
	Male	128	43 (43.9)	36 (32.1)	49 (44.5)		
Select the smile design you find more aesthetically pleasing in CASE:1	AI Preferred	100	31 (31.6)	38 (33.9)	31 (28.2)	0.863	0.65
	Manual Preferred	220	67 (68.4)	74 (66.1)	79 (71.8)		
Select the smile design you find more aesthetically pleasing in CASE:2	AI Preferred	98	32 (32.7)	32 (28.6)	34 (30.9)	0.416	0.812
	Manual Preferred	222	66 (67.3)	80 (71.4)	76 (69.1)		
Select the smile design you find more aesthetically pleasing in CASE:3	AI Preferred	126	34 (34.7)	48 (42.9)	44 (40)	1.487	0.476
	Manual Preferred	194	64 (65.3)	64 (57.1)	66 (60)		
Select the smile design you find more aesthetically pleasing in CASE:4	AI Preferred	127	39 (39.8)	44 (39.3)	44 (40)	0.013	0.994
	Manual Preferred	193	59 (60.2)	68 (60.7)	66 (60)		
% preference to AI	0	80	30 (30.6)	25 (22.3)	25 (22.7)	11.231	0.189
	25	89	17 (17.3)	38 (33.9)	34 (30.9)		
	50	111	39 (39.8)	32 (28.6)	40 (36.4)		
	75	20	7 (7.1)	8 (7.1)	5 (4.5)		
	100	20	5 (5.1)	9 (8)	6 (5.5)		

The bold values in Table 2 represent p-values and indicate statistically significant differences among the groups.

**Fig. 5:**
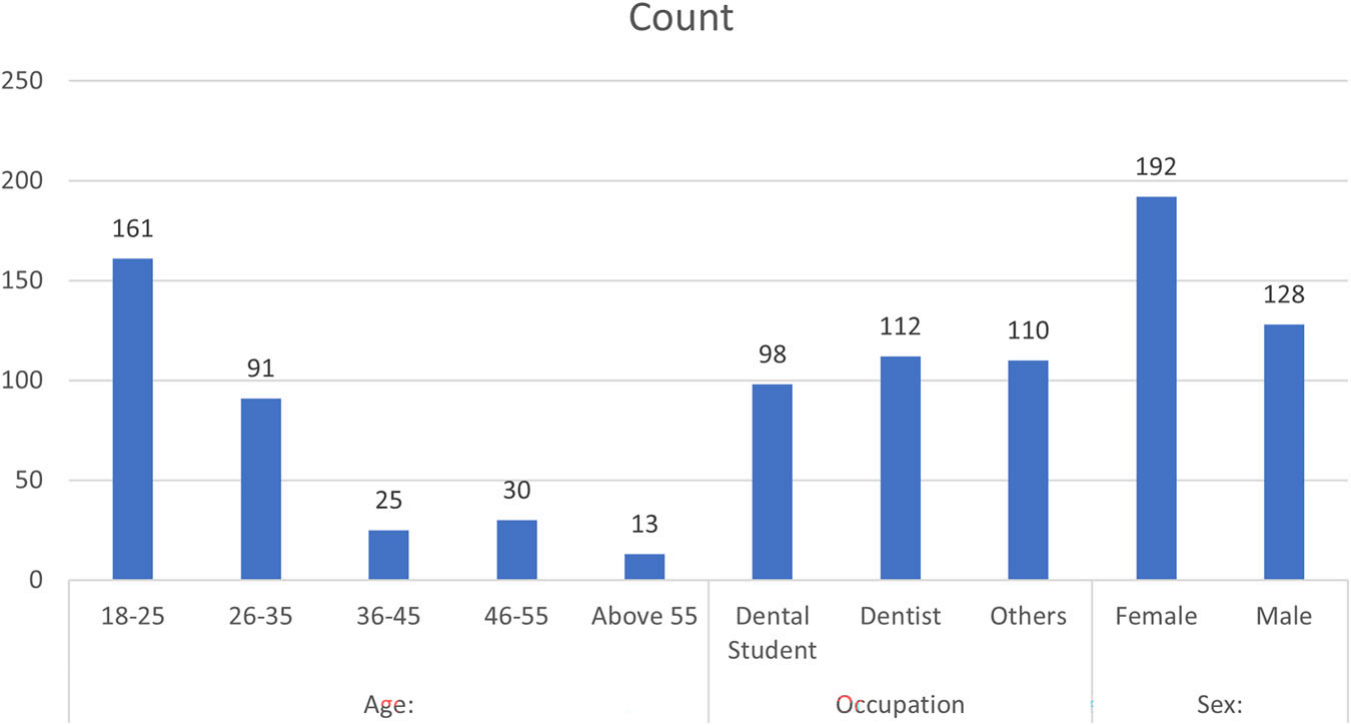
Distribution of study participants based on age group, occupation, and sex.

The distribution of participants across different age groups was significantly associated with occupation (χ² = 99.618, *p* < 0.001). Among those aged 18–25 years, 86.7% were dental students, whereas only 33.9% of dentists and 34.5% of others fell into this age range. In the 26–35 years age group, the proportion of dental students dropped to 13.3%, whereas 39.3% of dentists and 30.9% of others belonged to this category. The trend continued in older age groups, with no dental students in the 36–45, 46–55, or above 55 categories, whereas the percentage of dentists and others gradually increased with age (Fig. 5).

With respect to sex distribution, 56.1% of females were dental students, compared with 67.9% of dentists and 55.5% of others (Fig. 5). Among males, 43.9% were dental students, 32.1% were dentists, and 44.5% fell into the ‘others’ category. However, the association between sex and occupation was not statistically significant (*χ*² = 4.442, *p* = 0.109).

**Fig. 6:**
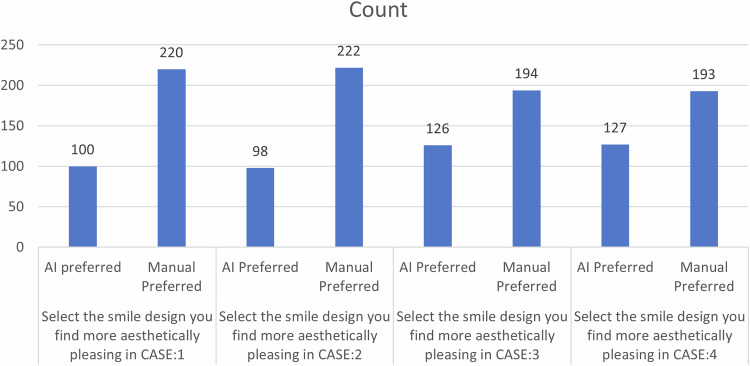
Comparison of participants’ preferences between AI-generated and manually designed smiles across four different cases.

In terms of aesthetic preference for smile designs, there was no significant difference in preference for AI-generated versus manually created designs across all four cases (Fig. 6). In Case 1, 31.6% of the dental students, 33.9% of the dentists, and 28.2% of the others preferred the AI-generated smile, with a nonsignificant chi-square value (χ² = 0.863, *p* = 0.65). Similar patterns were observed for Case 2 (*χ*² = 0.416, *p* = 0.812), Case 3 (*χ*² = 1.487, *p* = 0.476), and Case 4 (*χ*² = 0.013, *p* = 0.994), indicating no statistically significant preference for AI or manual design based on occupation.

**Fig. 7:**
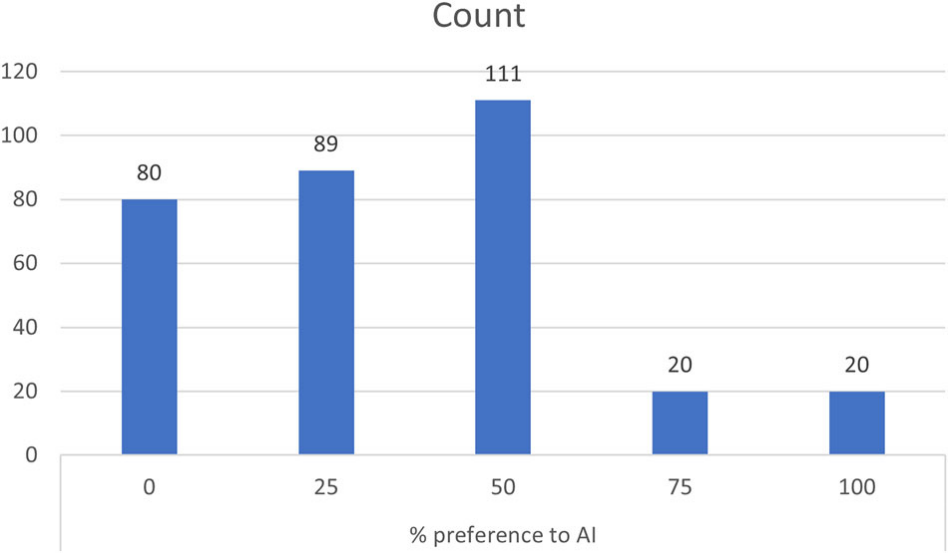
Distribution of participants based on percentage preference toward AI-designed smiles.

When the overall preference for AI-generated smile designs was analysed, 30.6% of the dental students, 22.3% of the dentists, and 22.7% of the others showed no preference for AI. Preference levels of 25%, 50%, 75%, and 100% AI-generated designs varied across the groups, but the association between AI preference and occupation was not statistically significant (χ² = 11.231, *p* = 0.189) (Fig. 7).

Table 3 shows the results of the statistical analysis comparing gender with age, occupation and aesthetic preference.

**Table 3:** Statistical analysis for comparison of gender with age, occupation and aesthetic preference.

	Categories	*N*	Sex:		Chi square	*P* value
			Female (*N* (%))	Male (*N* (%))		
Age:	18–25	161	100 (52.1)	61 (47.7)	3.996	0.407
	26–35	91	49 (25.5)	42 (32.8)		
	36–45	25	17 (8.9)	8 (6.2)		
	46–55	30	20 (10.4)	10 (7.8)		
	Above 55	13	6 (3.1)	7 (5.5)		
Occupation	Dental Student	98	55 (28.6)	43 (33.6)	4.442	0.109
	Dentist	112	76 (39.6)	36 (28.1)		
	Others	110	61 (31.8)	49 (38.3)		
Select the smile design you find more aesthetically pleasing in CASE:1	AI preferred	100	58 (30.2)	42 (32.8)	0.242	0.622
	Manual Preferred	220	134 (69.8)	86 (67.2)		
Select the smile design you find more aesthetically pleasing in CASE:2	AI preferred	98	51 (26.6)	47 (36.7)	3.729	0.053
	Manual Preferred	222	141 (73.4)	81 (63.3)		
Select the smile design you find more aesthetically pleasing in CASE:3	AI preferred	126	73 (38)	53 (41.4)	0.369	0.544
	Manual Preferred	194	119 (62)	75 (58.6)		
Select the smile design you find more aesthetically pleasing in CASE:4	AI preferred	127	74 (38.5)	53 (41.4)	0.263	0.608
	Manual Preferred	193	118 (61.5)	75 (58.6)		
% preference to AI	0	80	53 (27.6)	27 (21.1)	5.657	0.226
	25	89	52 (27.1)	37 (28.9)		
	50	111	65 (33.9)	46 (35.9)		
	75	20	14 (7.3)	6 (4.7)		
	100	20	8 (4.2)	12 (9.4)		

The distribution of participants across different age groups was not significantly associated with sex (χ² = 3.996, p = 0.407). Among those aged 18--25 years, 52.1% were female, and 47.7% were male. Similarly, the proportions of females and males across older age groups did not significantly differ. In the 26–35 years age group, 25.5% were female, and 32.8% were male. The percentage of females was greater in the 36-45 (8.9%) and 46-55 (10.4%) age groups than that of males (6.2% and 7.8%, respectively). However, in the above 55 years of age, males (5.5%) slightly outnumbered females (3.1%).

With respect to occupation, 28.6% of the dental students were female, whereas 33.6% were male. Among the dentists, 39.6% were female, whereas 28.1% were male. In the ‘others’ category, 31.8% were female, and 38.3% were male. However, the association between occupation and sex was not statistically significant (*χ*² = 4.442, *p* = 0.109).

In terms of the aesthetic preference for the smile design, there was no statistically significant difference between males and females in any of the cases. In Case 1, 30.2% of females and 32.8% of males preferred the AI-generated smile (*χ*² = 0.242, *p* = 0.622). In Case 2, a slightly greater proportion of males (36.7%) preferred the AI-generated smile than did females (26.6%), but this difference did not reach statistical significance (χ² = 3.729, *p* = 0.053). Similarly, in Case 3 (*χ*² = 0.369, *p* = 0.544) and Case 4 (*χ*² = 0.263, *p* = 0.608), there was no significant difference in the preference for AI-generated smiles between males and females.

When the overall preference for AI-generated smile designs was analysed, 27.6% of females and 21.1% of males had no preference for AI. The proportions of participants who preferred AI at 25%, 50%, 75%, and 100% varied between males and females, but the association between AI preference and sex was not statistically significant (χ² = 5.657, *p* = 0.226).

## Discussion

Facial aesthetics are a central concern in modern prosthodontics, as an aesthetically pleasing smile contributes significantly to self-esteem, psychosocial well-being, and interpersonal communication [26]. With advancements in digital dentistry, artificial intelligence (AI) has emerged as a transformative tool, offering automated, consistent, and efficient smile design workflows [8, 27]. AI-based systems can rapidly process facial and dental data to generate smile simulations that adhere to predefined aesthetic standards, potentially reducing subjectivity and chair-side time [21]. However, despite these advancements, the integration of AI into dental aesthetics remains a subject of ongoing research and clinical debate. This study sought to evaluate the aesthetic preferences for AI-generated versus conventionally crafted digital smile designs among dentists, dental students, and laypersons. By using paired comparisons of identical clinical situations, the study ensured standardization. The exclusive use of female subjects, although a limitation, was intended to minimize facial variability and align with documented aesthetic benchmarks in the female smile design literature. Exo-CAD software was used for both AI and manual design, and responses were gathered via a structured online survey. In line with research by Jain et al. [18] and Omar et al., [2] who highlighted the indispensable value of human artistic judgment and emotional perception in aesthetic evaluations, the results revealed a statistically significant preference for manually designed smiles across all participant groups. Although Smilecloud and Exo-CAD, two AI-driven smile design platforms, provide high levels of accuracy and reproducibility, participant preferences clearly highlight these systems’ shortcomings in simulating real facial dynamics and emotional context [2, 19].

In the assessment of aesthetic preferences for smile design, Case 3 and Case 4 presented the highest preference for AI-generated designs, with 39.4% and 39.7% of participants selecting the AI-generated smile, respectively. In contrast, Case 1 and Case 2 had lower AI preference rates at 31.2% and 30.6%, indicating a stronger inclination toward manually created designs in these cases. This variation in preference raises an important question for further exploration. Why did AI-generated smiles in Cases 3 and 4 receive relatively greater acceptance than those in Cases 1 and 2? Several factors could contribute to this trend:

1. *Tooth Proportions and Symmetry:* AI algorithms might have created smiles in Cases 3 and 4 that were more proportionate or symmetrical, aligning with the golden ratio principles often associated with smile esthetics.
2. *Tooth Shape and Arch Form:* The AI-generated designs in Cases 3 and 4 may have incorporated subtle differences in incisal edge morphology or arch form that resonated better with participants.
3. *Lip Dynamics and Soft Tissue Harmony:* Cases 3 and 4 might have presented liptooth relationships in which the AI managed to optimize more effectively than those in Cases 1 and 2 did.
4. *Perception of Naturalness:* Manual designs in Cases 1 and 2 may have retained features that appeared more natural, making them preferable over AI-modified versions.

A smile’s visual attractiveness is subjective and multifaceted and impacted by factors such as lip movement, facial symmetry, and individual personality traits—factors that AI algorithms are still learning to properly interpret [16]. This emphasizes how crucial therapeutic expertise and creative intuition are in producing results that are both patient-specific and harmonious. However, one should not overlook the promise of AI in the context of dental aesthetics. Its ability to streamline workflows, enhance reproducibility, and reduce human error makes it a valuable supplement in clinical practice [28, 29]. As AI models continue to evolve through deep learning and exposure to diverse datasets, their capacity to emulate human-like artistic outputs is expected to improve [30]. Future innovations may also integrate facial expression analysis and emotional feedback loops to refine design personalization.

Interestingly, our findings highlight an important implication for future research: the possibility of hybrid smile design protocols that blend the strengths of AI automation with manual refinements. Such models could leverage AI to generate preliminary designs, which can then be artistically enhanced by experienced clinicians, thereby combining efficiency with individualization. This aligns with emerging concepts in aesthetic dentistry that advocate for collaborative intelligence, where machines assist but do not replace human decision-making [31]. Furthermore, the study identifies key areas where research remains limited. For example, while aesthetic preferences were evaluated, longitudinal outcomes—such as patient satisfaction posttreatment and retention of aesthetic appeal—were not explored. Additionally, incorporating psychometric scales and standardized aesthetic indices (such as the pink and white aesthetic scores) could offer a more objective framework for future evaluations [25].

Our findings are in line with those of Ceylan et al. [19], who reported that manually crafted smiles were generally favoured, although AI designs were accepted in cases of facial symmetry. Similar to the observations of Jain et al. [18], our study reiterates that AI lacks the nuanced perception and creative touch offered by human clinicians. Unlike Babaei et al. [23], who reported no significant difference in preferences, our results emphasize a statistically consistent tilt towards manual designs, suggesting that cultural and regional aesthetic values may influence perceptions.

To the best of our knowledge, this is one of the first large-scale Indian studies to directly compare AI-generated versus manually crafted smile designs via real clinical cases and a diverse participant pool. It uniquely includes input from both professionals and laypersons, offering a holistic view of aesthetic perception in prosthodontics.

These findings suggest cautious optimism toward AI in aesthetic dentistry. While automation offers consistency and speed, the limitations in replicating nuanced facial dynamics and emotional harmony remain prominent, as also reported by Omar et al. [2]. and Jain et al. [18]. Our results reinforce the idea that AI-generated designs are more accepted in symmetrical or less complex cases (as Ceylan et al. [19]., observed), indicating the potential for partial integration in standard cases while preserving manual craftsmanship for high-aesthetic-demand scenarios. This finding supports the evolving clinical framework toward hybrid models, where AI provides baseline designs, later refined by clinicians—a model aligned with ‘collaborative intelligence’ in digital prosthodontics.

### Limitations

The use of an online survey method, while advantageous for reaching a wide and diverse population, introduces inherent limitations. Notably, the lack of control over respondent selection may lead to response bias. Unlike traditional survey methods that target specific subpopulations, online platforms do not allow for strict control over participant representativeness, and we acknowledge that this may influence generalizability. Additionally, the evaluation of smile aesthetics is subjective and can be influenced by device screen quality and ambient viewing conditions, which are not standardized across participants. Despite these limitations, this study provides valuable preliminary insights into the clinical acceptability of AI in smile design.

## Conclusion

This study highlights the evolving role of artificial intelligence in aesthetic dentistry and its current limitations in fully replicating the nuanced perceptions of beauty held by dental professionals and the general public. Despite the growing precision and efficiency offered by AI-driven smile design software, participants consistently preferred manually designed smiles across all demographics—dentists, dental students, and laypersons. According to these results, artificial intelligence (AI) can be a useful tool for optimizing digital workflows, but it is still unable to fully replace humans in terms of their capacity to recognize facial harmony, emotion, and uniqueness in smile aesthetics. In prosthodontic treatment planning, the preference for manual designs serves as further evidence of the ongoing significance of clinical knowledge, creative intuition, and patient-specific customization.

However, these conclusions must be viewed in light of methodological limitations, particularly the use of an online survey with potential response bias and uncontrolled viewing conditions. Future research with controlled clinical environments, objective aesthetic indices, and longitudinal follow-up is necessary to further validate the role of AI in prosthodontic aesthetics. Future developments should concentrate on incorporating hybrid models that blend the effectiveness of AI with clinician-guided improvements. Further research using longitudinal evaluations and a variety of aesthetic metrics is also necessary to confirm the long-term usability and functional results of AI-generated designs. In conclusion, the clinician’s skill in creating aesthetically pleasing and emotionally impactful smiles is enhanced by artificial intelligence rather than replaced.

## Data Availability

The datasets generated and/or analyzed during the current study are available from the corresponding author on reasonable request.
